# Getting physicians to open the survey: little evidence that an envelope teaser increases response rates

**DOI:** 10.1186/1471-2288-12-41

**Published:** 2012-03-31

**Authors:** Jeanette Y Ziegenfuss, Kelly Burmeister, Katherine M James, Lindsey Haas, Jon C Tilburt, Timothy J Beebe

**Affiliations:** 1 Mayo Clinic 200 First Street Southwest Rochester, Minnesota, 55905, USA; 2 Children's Hospital Boston, 300 Longwood Ave, Boston, MA 02115, USA; 3 Health Care Policy & Research - Mayo Clinic, 200 First Street Southwest, Rochester, MN 55905, USA

**Keywords:** Survey methods, Response rates, Physician surveys

## Abstract

**Background:**

Physician surveys are an important tool to assess attitudes, beliefs and self-reported behaviors of this policy relevant group. In order for a physician to respond to a mailed survey, they must first open the envelope. While there is some evidence that package elements can impact physician response rates, the impact of an envelope teaser is unknown. Here we assess this by testing the impact of adding a brightly colored "$25 incentive" sticker to the outside of an envelope on response rates and nonresponse bias in a survey of physicians.

**Methods:**

In the second mailing of a survey assessing physicians' moral beliefs and views on controversial health care topics, initial nonrespondents were randomly assigned to receive a survey in an envelope with a colored "$25 incentive" sticker (teaser group) or an envelope without a sticker (control group). Response rates were compared between the teaser and control groups overall and by age, gender, region of the United States, specialty and years in practice. Nonresponse bias was assessed by comparing the demographic composition of the respondents to the nonrespondents in the experimental and control condition.

**Results:**

No significant differences in response rates were observed between the experimental and control conditions overall (p = 0.38) or after stratifying by age, gender, region, or practice type. Within the teaser condition, there was some variation in response rate by years since graduation. There was no independent effect of the teaser on response when simultaneously controlling for demographic characteristics (OR = 0.875, p = 0.4112).

**Conclusions:**

Neither response rates nor nonresponse bias were impacted by the use of an envelope teaser in a survey of physicians in the United States.

## Background

Health services researchers are dependent on physicians' participation in surveys in order to assess provider attitudes, beliefs and self-reported behaviors such as guideline adherence. There is some evidence that physician response rates have been falling in more recent years [1,2]. While low response rates are known to reduce statistical power and result in higher costs per completed survey, the impact of low response rates on nonresponse bias may not be as strong as previously thought [3]. Nevertheless, exploring methods to increase response rates in surveys of physicians is an important area of study, one that was recently called for in a review of the physician response literature by VanGeest et al. [4] In November of 2010, the National Cancer Institute convened a Provider Survey Methods Workshop where a research agenda for the field was developed, underscoring the broad attention to surveying this population with specific attention to methods designed to enhance participation. Due to the growing evidence that response rate is not necessarily correlated with response bias [3] these calls simultaneously call for systematic examination of nonresponse bias as is undertaken herein.

For an individual to respond to a mailed survey a series of steps must occur - not the least of which is opening the envelope in which the survey is sent. While this may seem trivial, for physicians it may be less so. There is evidence that physicians receive a large amount of materials (including surveys) in the mail [5] thus taking this important first step is far from guaranteed. In a qualitative study asking nonresponding general practitioners why they did not respond, fully 34% said that the questionnaire "got lost in a pile of paper work." [6] An additional barrier to obtaining survey responses from physicians is the potential presence of gatekeepers, or individuals who screen physicians' mail and pass on only those materials which, in the absence of any clarifying information about their contents, are deemed "important" by a third party. While this extent of gatekeeping has not been empirically documented, it has been hypothesized to put a downward pressure on response rates [4,7-9]. In preparation for our own earlier work, we have anecdotal evidence that this is the case. We conducted informal focus groups in a physician population about potential issues in responding to surveys; not opening one's mail was a cited barrier [9].

One way to motivate the person who would regularly be responsible for opening mail (either the potential responders or the gatekeeper if indeed they do exist as hypothesized) to open an envelope is with a "teaser" on the outside of the envelope itself. This teaser would entice or suggest to the recipient that it is worth their time to open the letter or package, lessening the chance that the envelope is "lost in a pile of paperwork". A recent meta-analysis of methods to increase response rate suggested that including an envelope teaser increased response rates more than three-fold [10]. This conclusion, however, was based on only one study with a sample size of 190 in which recipients were assigned to an envelope teaser condition (i.e. a stamp on the envelope reading "Did you know you were entitled to more money?") or a control group with no such stamp [11]. While this finding is suggestive, the small sample size does not support a strong inference, nor can the findings be generalized to a physician population. Moreover, the current context of mailed surveys is likely quite different more than a decade after this initial publication.

More generally, however, there is evidence that other elements of packaging can significantly affect response rates in physician surveys (for review see: Kellerman & Herold [1]). For example, Asch and Christakis [12] manipulated the envelope so that the letter appeared from the University Medical School or the Veterans Affairs Hospital. Response rates were significantly higher for the latter envelope, suggesting that aspects of the envelope itself can matter and that respondents were more likely to open an envelope from the Veteran Hospital than from the University Hospital as conveyed on the outside of the envelope itself. Moreover, in the executive summary from the National Cancer Institute's provider methods workshop the importance of engaging physicians with the envelope itself was cited due to the observation that the decision about whether to open envelopes may be made "in seconds"[13].

Here we report results of a randomized study testing the assertion that an envelope teaser could increase response rates among a nationally representative physician population receiving a mailed questionnaire. This manipulation occurred in the second wave mailing to initial survey nonrespondents.

## Methods

### Samples and procedures

The envelope teaser experiment was embedded in a survey assessing physicians' moral beliefs and views on controversial healthcare topics, the substantive methods and findings of which are reported elsewhere [14]. In May 2009, we mailed a self-administered, 8-page survey to 2,000 practicing U.S. physicians ages 65 and younger and representing all specialties. Our random sample was selected from the AMA Physician Masterfile.

The first mailing of the survey included a book ("The Quotable Osler") as an incentive and promised an additional $25 check to all respondents. Nonrespondents to the first mailing (n = 1,250) were sent another copy of the survey six weeks later with the same promised $25 check upon survey completion. In this second wave mailing, we randomized nonrespondents to receive either an envelope with a brightly colored sticker reading "$25 incentive" (n = 630) or an envelope with no sticker (n = 620). This study was approved by the Mayo Clinic Institutional Review Board.

### Analysis

Overall response rates between the teaser and control groups were compared using a Chi-square goodness-of-fit test. Response rates were also compared by gender, age, geographic region, years since graduation from medical school, and practice type (i.e. office-based practice or full time hospital staff). Multivariate analysis was undertaken to isolate the impact of the teaser, controlling for available demographic and practice characteristics. The relative representativeness of responders compared to nonresponders was also compared with respect to demographic characteristics to further assess nonresponse bias across conditions.

## Results

Overall response rates did not differ significantly comparing the teaser (15.9%) and control (14.2%) groups (p = 0.38) (see Table 1). Within the teaser group there were no overall differences in response rates after stratifying on age, gender, region or practice type (office or hospital), indicating no differential nonresponse bias. However, this was not the case for years since graduation. Among physicians in the teaser group, a higher response rate was observed in those having graduated 5-9 years ago (27.1%) compared to those who graduated 10-19 years ago (11.6%) (p = 0.03), indicating that there may be a small nonresponse bias with respect to years since graduation when a teaser is used, however this relationship did not hold up in the multivariate analysis (results not shown). There were no differences in response rates by demographic or practice characteristics within the control condition for any of the examined characteristics. Moreover, when controlling simultaneously for all of the demographic and practice characteristics, the teaser did not predict response (OR = 0.875, p = 0.4112) (results not shown).

**Table 1:** Response Rates Between and Within Groups

	No Response (N = 1062)	Response (N = 188)	Total (N = 1250)	p value
**Age Group**				0.4568
< 34	80 (7.5%)	12 (6.4%)	92 (7.4%)	
34-44	314 (29.6%)	61 (32.4%)	375 (30.0%)	
45-54	375 (35.3%)	56 (29.8%)	431 (34.5%)	
55-64	272 (25.6%)	53 (28.2%)	325 (26.0%)	
65+	21 (2.0%)	6 (3.2%)	27 (2.2%)	
**Gender**				0.7014
Missing	42 (.%)	3 (.%)	45	
Female	300 (29.4%)	57 (30.8%)	357 (29.6%)	
Male	720 (70.6%)	128 (69.2%)	848 (70.4%)	
**Office vs. Hospital**				0.9240
Missing	2 (.%)	0 (.%)	2	
Office Based	972 (91.7%)	172 (91.5%)	1144 (91.7%)	
Hospital Based	88 (8.3%)	16 (8.5%)	104 (8.3%)	
**Census Region**				0.4286
West	233 (21.9%)	40 (21.3%)	273 (21.8%)	
Midwest	199 (18.7%)	41 (21.8%)	240 (19.2%)	
Northeast	241 (22.7%)	39 (20.7%)	280 (22.4%)	
Other	20 (1.9%)	7 (3.7%)	27 (2.2%)	
South	369 (34.7%)	61 (32.4%)	430 (34.4%)	
**Years since graduation**				0.0713
< 5	7 (0.7%)	1 (0.5%)	8 (0.6%)	
5-9	86 (8.1%)	26 (13.8%)	112 (9.0%)	
10-19	380 (35.8%)	58 (30.9%)	438 (35.0%)	
20+	589 (55.5%)	103 (54.8%)	692 (55.4%)	
**Sticker**				0.4062
No	532 (50.1%)	88 (46.8%)	620 (49.6%)	
Yes	530 (49.9%)	100 (53.2%)	630 (50.4%)	

## Discussion

Adding a teaser sticker to the outside of an envelope in a mailed survey did not significantly increase response rates among a nationally representative sample of physicians in the United States. However, because the relative cost of adding an envelope teaser is minimal, the evidence that it introduces nonresponse bias small, and given the sparse existing literature on envelope teasers, future work in this area is warranted. Specifically, wording variations on the teaser sticker itself, the incentive amount and type (monetary vs. nonmonetary and guaranteed vs. lottery) should be further tested in a physician population. It is possible that the teaser for a $25 incentive offer was insufficient to change behaviors in this population of physicians, as the findings of Keating et al. [15], who found significantly higher response rates in a survey of physicians when a prepaid check for $50 was issued versus a prepaid check for $20, would suggest.

This study has several limitations. One of the mechanisms through which the envelope teaser is hypothesized to work is through increasing the chance that a physician gatekeeper would pass the survey along to the potential respondent, thereby increasing the likelihood of response. The present study cannot determine if there was a differential response to the envelope teaser by the presence or absence of a gatekeeper as it did not explicitly measure the presence or absence of a gatekeeper. Future research should work toward moving beyond the proposed existence and impact of gatekeepers to a documentation of this extent that gatekeeping actually impacts survey response. Once this is determined, the mechanism by which something like a teaser functions with or without gatekeeping can be elucidated. Because the present study provided a manipulation in the follow-up to a survey, its findings cannot be generalized to an initial mailing as all of the responders in this experiment had already not responded to an initial request. In order to better understand the true potential of envelope teasers, and given their low cost, their value should be tested in initial mailings as well as later mailings. Finally, the extent that these findings can be generalized beyond physicians in the United States is unknown.

## Conclusion

In conclusion, even though neither response rates nor nonresponse bias were impacted by the use of an envelope teaser in a survey of physicians in the United States, it is important that health survey methodologists continue to respond to the calls of Cull et al. [16], Kellerman and Harold [1], Klabunde et al. [13], McMahon et al. [17], and VanGeest and Johnson [4] by continuing to further test methods of enhancing survey participation among elite populations such as physicians while simultaneously examining nonresponse bias. While there is mounting evidence that there is no linear relationship between response rate and nonresponse bias it is important to consider the potential for nonresponse bias in each survey context [3]. Otherwise, the physician perspective may not be adequately represented in debates and issues germane to the practice of medicine or to the realm of health care reform.

## Competing interests

The authors declare that they have no competing interests.

## Authors' contributions

JZ wrote the manuscript, supervised the analysis and led the interpretation of findings. KB and KMJ assisted with the writing and interpretation of findings. LH performed the analysis. JCT led the study in which the present study was embedded and designed this study. TJB oversaw data collection and interpretation of findings. All authors approved a final version of the manuscript.

## Pre-publication history

The pre-publication history for this paper can be accessed here:

http://www.biomedcentral.com/1471-2288/12/41/prepub
